# Mars Potato Cultivation: Analysis, Challenges, Sustainable Scientific Conceptions

**DOI:** 10.3390/life16020281

**Published:** 2026-02-05

**Authors:** Bohao Yang, Yunjiang Liang

**Affiliations:** College of Agriculture, Yanbian University, Yanji 133002, China; 1234120998@ybu.edu.cn

**Keywords:** Martian agriculture, potato cultivation, soil remediation technologies, Bioregenerative Life Support Systems (BLSS)

## Abstract

As human space exploration advances towards establishing sustainable Martian habitats, achieving autonomous food production is a critical requirement. The potato (*Solanum tuberosum* L.), with its notable environmental resilience and nutritional efficiency, is a prime candidate crop. This study develops a conceptual framework for Martian potato cultivation by systematically analyzing the profound disparities between Martian conditions and plant physiology. We identify and evaluate seven fundamental challenges: atmospheric composition, extreme temperatures, water scarcity, soil properties, nutrient deficiencies, absent microbiota, and radiation/gravity effects. To address these challenges, we propose a phased, testable roadmap comprising four stages: (I) screening and bio-engineering of multi-stress-tolerant potato genotypes; (II) phased domestication via Earth-based analog experiments to define adaptability thresholds; (III) deployment of a controlled cultivation module within a Martian habitat, integrating targeted technological interventions; and (IV) conceptual exploration of extra-habitat agricultural potential. The primary contribution of this work is a structured set of hypotheses and key performance indicators for each stage, translating visionary goals into a defined research agenda to guide future empirical work in extraterrestrial agronomy.

## 1. Introduction

Human exploration of Mars has never ceased. As planetary exploration objectives have transitioned from short-term exploration to permanent settlement, the establishment of Bioregenerative Life Support Systems (BLSS) has become a central challenge in deep space exploration. However, both the long-term Mars residency missions required for establishing BLSS and the intrinsic goal of closed-loop food regeneration within BLSS necessitate achieving certain levels of crop production.

While discussions on Martian colonization persist, and some studies have demonstrated preliminary findings such as plant growth in Martian soil simulants [1] or the importance of genotype-soil interactions for salinity tolerance [2], the current body of research remains fragmented. A significant gap exists in the literature: the absence of a comprehensive, systems-level framework that integrates the disparate challenges of Martian agriculture—from plant physiology to environmental engineering—into a coherent, phased roadmap for a specific candidate crop.

The potato has long been regarded as a promising candidate for space exploration [3,4]. Its strong environmental tolerance and broad adaptability enable growth in diverse habitats, ranging from Arctic regions to tropical zones at elevations of 0–4000 m, including areas with extreme climatic and soil conditions [5,6]. Additionally, studies reveal that potatoes maintain normal growth under low-gravity and high-radiation conditions [7]. Compared to other crops, potatoes offer higher nutritional value [8], require less land area and water for equivalent yields [9], and ensure genetic stability through asexual propagation via tubers. This paper therefore focuses on proposing a scientific framework for sustainable potato cultivation on Mars, providing both theoretical foundations and technical pathways for Martian agriculture.

This study therefore aims to address this gap by developing a conceptual framework for sustainable potato cultivation on Mars. The novelty of our work lies in its synthesis of knowledge across disciplines to formulate a central, testable hypothesis: that a phased approach, prioritizing genetic adaptation alongside targeted technological interventions, can define a viable pathway towards this goal. To rigorously evaluate this hypothesis, we pursue three specific objectives: (1) to systematically quantify the disparities between Martian conditions and potato growth requirements; (2) to formulate a four-stage conceptual roadmap with defined milestones; and (3) to establish Key Performance Indicators (KPIs) that serve as benchmarks for the future empirical validation of each stage. The primary outcome of this synthesis is a structured research agenda designed to translate a high-level conception into a sequence of grounded, testable research priorities.

The structure of the paper is as follows: Section 2 examines Martian environmental conditions and soil requirements for potato cultivation. Section 3 compares these parameters to identify key challenges. Section 4 details the proposed implementation strategy for Martian potato farming. Finally, Section 5 concludes the study and outlines potential future research directions for optimization.

## 2. Natural Conditions of Mars and Requirements for Potato Growth

A foundational step in conceptualizing agriculture on Mars is to move beyond a simple listing of environmental challenges. This section establishes a hierarchical framework by first synthesizing the parametric disparities between Martian conditions and potato physiology and then critically evaluating these parameters based on their inherent modifiability. This analysis allows us to stratify the challenges into those that define absolute boundary conditions and those that can be systematically engineered, thereby providing the logical foundation for the phased roadmap developed in Section 4.

The parametric disparities detailed in Table 1 necessitate a fundamental stratification of constraints based on their inherent modifiability, which in turn dictates the essential architecture of any cultivation system.

Globally immutable parameters—specifically atmospheric pressure, solar radiation, and gravity—constitute fixed boundary conditions of the Martian planetary environment. These cannot be engineered at a meaningful scale. Their immutability establishes a non-negotiable prerequisite: a fully engineered, shielded, and pressurized growth habitat is an absolute requirement. This conclusion directly prioritizes foundational research avenues: the development of containment and radiation-shielding technologies and the study of plant physiology and adaptation under hypogravity.

In contrast, locally manageable parameters encompass temperature, atmospheric composition (within a contained volume), water availability, and soil properties (pH, structure, nutrients, and microbiology). While achieving control over these parameters to meet plant requirements is energetically and logistically formidable, the core scientific and engineering principles—such as thermal regulation, gas scrubbing, irrigation, soil amendment, and fertilization—are well-established. The primary challenge for Mars shifts from one of fundamental feasibility to one of systems integration, reliable operation, and resource optimization under extreme constraints.

This hierarchical analysis is not merely an academic exercise; it provides the foundational logic for the staged research roadmap proposed in this paper. The immutable constraints justify initial research focused on screening and bio-engineering genotypes for radiation and hypogravity tolerance and define the required performance characteristics of the cultivation habitat. The manageable parameters delineate the specific engineering and agronomic interventions that must be developed and integrated within that habitat. By clearly distinguishing between what must be accepted as a fixed boundary condition and what can be engineered, this analysis transforms a list of problems into a structured set of design requirements and research priorities.

## 3. A Hierarchical Framework of Challenges

**Figure 1:**
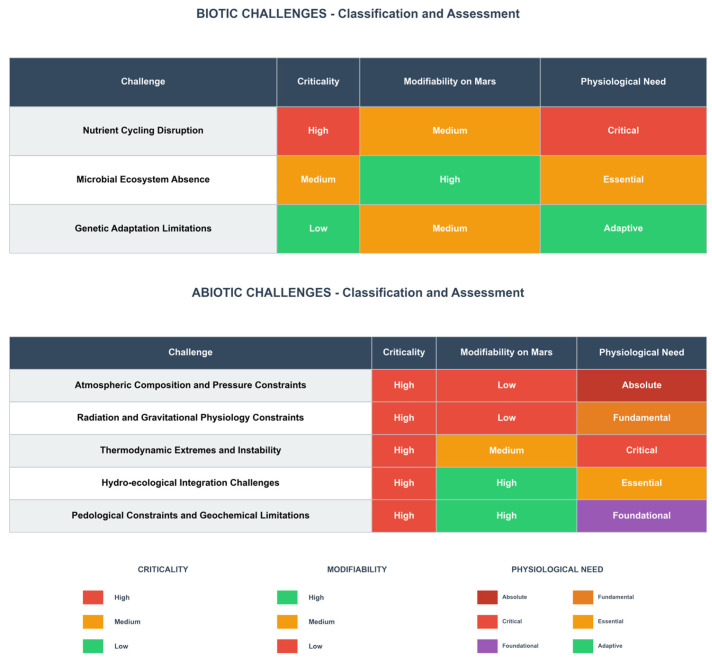
Classification of Challenges for Martian Potato Cultivation.

Building upon the parametric constraints defined in Section 2, this section establishes a hierarchical framework to classify challenges as either biotic or abiotic, subsequently ranking them by criticality (High, Medium, Low) through an integrated assessment of their modifiability on Mars and physiological imperative for the plant, as synthesized in Figure 1. This framework structures the subsequent analysis into biotic (Section 3.1) and abiotic challenges (Section 3.2), wherein challenges are examined in descending criticality order to elucidate their physiological mechanisms and formulate testable hypotheses, thereby establishing the logical foundation for the development roadmap in Section 4.

### 3.1. Biotic Challenges

Biotic challenges encompass biological processes and interactions critical for sustainable cultivation systems. These factors present fundamental barriers to establishing functional agricultural ecosystems under Martian conditions.

#### 3.1.1. Nutrient Cycling Disruption

The Martian system exhibits a complete breakdown in biogeochemical cycling mechanisms. The regolith’s inherent sterility and lack of organic matter prevent essential nutrient transformations, creating a biochemically inert growth medium. This disruption manifests most critically in the nitrogen cycle, where the absence of microbial mineralization pathways locks existing nutrients in biologically unavailable forms.

The phosphorus cycle is equally compromised by the alkaline soil conditions, which precipitate plant-available phosphates into insoluble compounds. Potassium, while present in mineral form, remains largely inaccessible due to the lack of weathering processes and organic chelating agents. This triple limitation of nitrogen fixation, phosphorus solubility, and potassium mobilization represents a fundamental barrier to autonomous nutrient cycling.

The physiological implications extend beyond mere nutrient deficiency. Plants exhibit compromised root architecture and reduced rhizosphere activation, further limiting their ability to access mineral nutrients. The absence of soil organic matter eliminates the cation exchange capacity necessary for nutrient retention, creating a scenario where even supplemented nutrients are prone to leaching in the coarse-textured regolith.

#### 3.1.2. Microbial Ecosystem Absence

The sterile Martian environment lacks the microbial foundation upon which terrestrial agriculture depends. This absence disrupts multiple ecosystem services: organic matter decomposition, pathogen suppression, soil structure formation, and nutrient transformation. The vacuum in microbial diversity eliminates the biological insurance that buffers terrestrial systems against environmental stresses.

The missing microbiome particularly impacts plant health through the absence of mycorrhizal associations. These symbiotic relationships normally enhance water and nutrient uptake efficiency by extending the effective root zone. Without these partnerships, plants must allocate more resources to root growth at the expense of yield, while simultaneously facing reduced access to immobile nutrients like phosphorus.

The lack of functional microbial communities also prevents the development of disease-suppressive soils. In terrestrial systems, complex microbial interactions naturally suppress pathogens through competition, antibiosis, and induced systemic resistance. The sterile Martian regolith offers no such protection, creating conditions where any introduced pathogen could proliferate unchecked.

#### 3.1.3. Hydro-Ecological Integration Challenges

The hydro-ecological system presents a critical interface where biological and physical constraints converge. Water availability transcends mere irrigation requirements, representing a fundamental determinant of physiological functionality. The extreme aridity of the Martian environment (atmospheric H_2_O < 0.01%) creates a vapor pressure deficit that disrupts stomatal regulation and compromises leaf-level gas exchange. This atmospheric desiccation is compounded by the regolith’s limited water retention capacity, where the absence of organic matter and clay minerals reduces moisture-holding capacity to approximately one-third of terrestrial agricultural soils.

The physiological implications extend beyond simple drought stress. The high salinity of Martian regolith (0.5–1.0 wt% chloride) establishes an osmotic gradient that further impedes water uptake, while the low atmospheric pressure reduces the boiling point of water, creating conditions where evaporative cooling becomes increasingly inefficient. This combination of factors threatens to disrupt thermal regulation mechanisms essential for maintaining enzymatic activity and membrane integrity.

Root architecture development faces additional constraints from the regolith’s physical properties. The limited porosity (<30%) and high bulk density (1.6–1.8 g/cm^3^) restrict root penetration and exploration, while the absence of stable soil aggregates reduces the rhizosphere’s capacity for water and gas exchange. These limitations collectively constrain the plant’s ability to access and utilize available moisture, creating a scenario where even technically sufficient water supplies may remain physiologically inaccessible.

#### 3.1.4. Genetic Adaptation Limitations

The biological constraints extend to fundamental genetic limitations in plant adaptation capacity. Current terrestrial potato varieties lack evolutionary exposure to Martian conditions, particularly the complex interplay of hypogravity (0.38 g), cosmic radiation (0.67 mSv/day), and altered atmospheric composition. This genetic gap manifests in inadequate stress response pathways and suboptimal physiological adaptations for the unique Martian environment.

The radiation environment presents particular challenges at the cellular level. Galactic cosmic rays and solar particle events generate DNA damage patterns distinct from terrestrial radiation exposures, requiring novel repair mechanisms that existing varieties may lack. Concurrently, the reduced gravity environment affects auxin transport and distribution, potentially disrupting tropic responses and source-sink relationships essential for tuber development.

The temporal scale of adaptation presents another fundamental constraint. While terrestrial crops have undergone millennia of selection pressure for Earth conditions, the rapid establishment of Martian agriculture necessitates accelerated adaptation through either intensive breeding or genetic engineering. This compression of evolutionary timescales creates significant challenges in developing varieties with sufficient resilience to the multifactorial stresses of the Martian environment.

The metabolic implications are particularly evident in carbon fixation pathways. The high CO_2_ environment (95.3%), while potentially beneficial for photosynthesis, may overwhelm existing regulatory mechanisms and lead to metabolic imbalances. Furthermore, the altered day-night cycle (24 h 39 m) and reduced light intensity could disrupt circadian rhythms and photosynthetic efficiency, requiring genetic adjustments that current varieties lack.

### 3.2. Abiotic Challenges

Abiotic factors constitute the fundamental physical and chemical parameters of the Martian environment that establish absolute boundary conditions for plant growth. These non-biological constraints require engineering solutions at the system level.

#### 3.2.1. Atmospheric Composition and Pressure Constraints

The Martian atmospheric environment presents a fundamental physiological barrier characterized by synergistic limitations of hypobaria and hypercapnia. With a surface pressure of approximately 0.6 kPa—less than 1% of Earth’s sea-level pressure—the near-vacuum conditions prevent maintenance of cellular turgor and disrupt stomatal regulation mechanisms essential for gas exchange. This pressure deficit creates an environment where liquid water stability is physiologically compromised, directly impacting hydraulic conductivity and nutrient transport.

The atmospheric composition, dominated by CO_2_ at 95.3%, creates a condition of extreme hypercapnia that inhibits key photosynthetic enzymes, including Rubisco activase. The critical absence of molecular oxygen (≤0.1% versus the required 18–21% for aerobic respiration) forces a transition to anaerobic metabolic pathways that are energetically inefficient for complex multicellular plants. This combination of hypobaria and hypercapnia induces complex gas exchange limitations that affect both photosynthetic efficiency and respiratory function.

The integrated physiological impact manifests most critically during tuber initiation, where soil oxygen partial pressure below 18 kPa can inhibit tuberization rates by over 40%. The root zone hypoxia exacerbates the effects of atmospheric hypoxia, creating a compounded stress that current potato genotypes are evolutionarily unprepared to tolerate.

#### 3.2.2. Thermodynamic Extremes and Instability

The Martian thermal environment presents a dual challenge of extreme absolute temperatures and unprecedented diurnal variations. With an average surface temperature of −63 °C and diurnal fluctuations exceeding 120 °C, the system operates outside the thermal kinetic windows for most plant enzymatic processes. The temperature regime consistently falls below the 7 °C threshold required for root development and frequently drops to −90 °C at night, inducing irreversible cellular damage through ice crystal formation.

The thermodynamic instability disrupts membrane fluidity and protein conformation, leading to loss of enzymatic activity and compromised membrane integrity. The extreme diurnal swing prevents metabolic acclimation, as the timescale of temperature change exceeds the plant’s capacity for biochemical adjustment through changes in membrane lipid composition or stress protein expression.

The thermal constraints extend to soil biogeochemical processes, where low temperatures reduce nutrient diffusion rates and microbial activity. The absence of atmospheric insulation creates a system where radiative heat loss dominates, making thermal management energetically prohibitive without significant technological intervention. The temperature extremes also affect water relations, with frozen soil conditions limiting water availability even when ice is physically present.

#### 3.2.3. Pedological Constraints and Geochemical Limitations

The Martian regolith exhibits fundamental pedological deficiencies that distinguish it from terrestrial agricultural soils. Unlike Earth’s well-developed soil profiles with distinct horizons (A-horizon: organic-rich surface layer, B-horizon: illuvial accumulation zone, C-horizon: parent material), the Martian surface consists primarily of minimally weathered basaltic material lacking stratigraphic differentiation. This immaturity results from limited hydrologic activity, minimal organic weathering processes, and the absence of biological mixing agents that drive soil formation on Earth.

The geochemical composition presents multiple constraints for plant growth. The alkaline conditions (pH 7.5–8.5) promote phosphorus fixation into insoluble calcium phosphate compounds, reducing bioavailability to less than 20% of typical agricultural soils. This alkalinity also creates cationic imbalances, particularly evident in the elevated calcium-magnesium ratio (Ca^2+^/Mg^2+^ > 10:1), which disrupts membrane stability and nutrient uptake efficiency. The high chloride content (0.5–1.0 wt%) induces both osmotic stress and specific ion toxicity, with experimental evidence demonstrating a 76% reduction in root viability at concentrations exceeding 0.3%.

Physical limitations further compound these chemical constraints. The regolith’s high bulk density (1.6–1.8 g/cm^3^) and limited porosity (<30%) restrict root penetration and reduce oxygen diffusion rates (ODR = 18 μg/cm^2^/min) below the critical threshold required for tuber formation (≥30 μg/cm^2^/min). The absence of clay minerals and organic matter eliminates the colloidal fraction necessary for maintaining structural stability and nutrient retention capacity. Magnetic properties distinct from terrestrial soils suggest unique iron mineralogy that may influence nutrient availability and soil-water interactions.

#### 3.2.4. Radiation and Gravitational Physiology Constraints

The unique space environment introduces fundamental biophysical challenges that affect plant growth at cellular and organismal levels. The radiation environment, characterized by galactic cosmic rays (GCR) and solar particle events (SPE), delivers chronic low-dose radiation (0.67 mSv/day) alongside acute high-energy particles. This radiation spectrum induces complex DNA damage patterns, including double-strand breaks that existing repair mechanisms may not efficiently address, potentially leading to genomic instability across generations.

The hypogravity conditions (0.38 g) disrupt fundamental biophysical processes evolved under Earth’s gravity. The reduced gravitational acceleration affects auxin transport and distribution, altering tropic responses and potentially compromising the gravitropic establishment essential for root architecture development. The altered hydrostatic pressure gradients may impact vascular function and cell wall expansion dynamics, potentially reducing mechanical strength and structural integrity. Studies have shown that low-gravity environments also have an impact on the transpiration rate of plants [37], further exacerbating the contradiction in water use.

## 4. A Conceptual Roadmap for Sustainable Potato Cultivation on Mars

**Figure 2:**
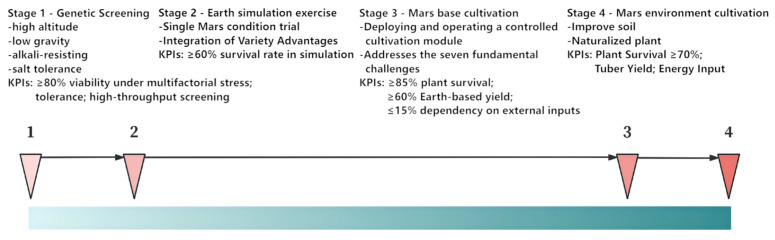
Four-Stage Development Timeline for Martian Potato Cultivation.

This section presents a conceptual framework comprising four developmental stages for establishing potato cultivation under Martian conditions. The proposed roadmap incorporates specific performance indicators at each phase to enable quantitative assessment of technological and biological milestones. Guided by principles of scientific rigor, operational feasibility, and long-term sustainability, this framework establishes large-scale production as the ultimate objective, with implementation progressing through the sequential stages illustrated in Figure 2.

**Figure 3:**
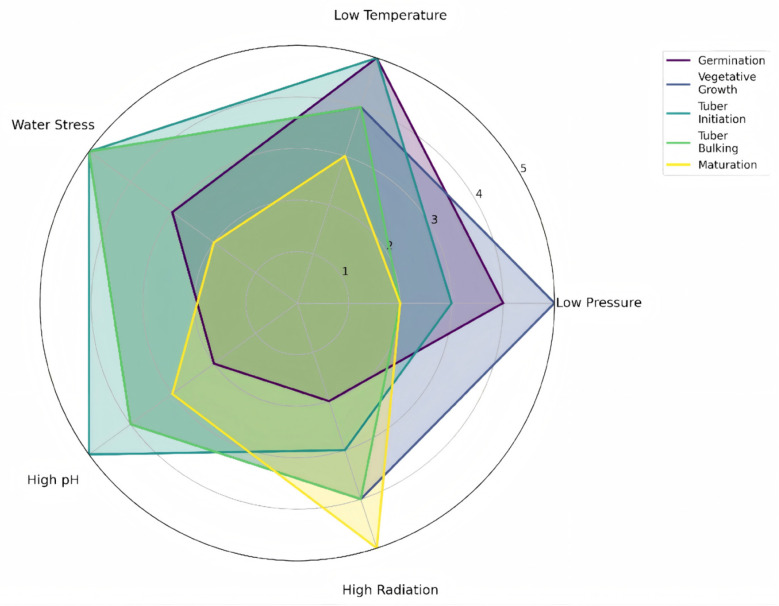
Radar Chart of the Sensitivity of Potato at Different Growth Stages to Key Environmental Parameters.

Physiological analysis reveals differential sensitivity to environmental stressors across distinct phenological stages, as quantified in Figure 3. Tuber initiation and bulking demonstrate particular vulnerability to hydrological stress and soil alkalinity, aligning with the challenges detailed in Section 3.1.3 and Section 3.2.3. Conversely, germination exhibits critical sensitivity to low-temperature stress, corresponding to the thermodynamic constraints outlined in Section 3.2.2. This physiological foundation justifies the staged approach: initial stages must identify genotypes exhibiting inherent resilience during critical developmental windows, particularly tuber initiation.

### 4.1. Stage 1 (Variety Screening)

The challenges associated with potato cultivation on Mars are multifaceted, and prioritizing environmental modification would entail prohibitive costs or technical infeasibility, particularly for immutable constraints such as Martian radiation and gravity. Consequently, the initial phase emphasizes the screening of potato varieties to identify germplasm with inherent resilience. The objective is to assemble genotypes demonstrating adaptation to high-altitude conditions (characterized by hypobaria and elevated radiation), tolerance to hypogravity, and resistance to alkalinity and salinity. High-altitude environments are analogous to Martian conditions due to attenuated atmospheric pressure and intensified solar radiation. Martian regolith contains sulfate, carbonate, chloride, and nitrate salts [38], rendering extreme salinity a primary abiotic stressor. Thus, integrating stress-tolerant genotypes with soil management strategies is imperative for achieving sustainable yields [2]. Varieties exhibiting adaptation to high altitude, hypogravity, alkalinity, and salinity collectively present the highest potential for Martian acclimatization. Complementary genetic engineering approaches may further enhance traits for cultivation in extreme environments.

Current research has documented significant advances in screening for salinity tolerance and high-altitude adaptability [39,40,41]. Although direct studies on alkaline tolerance are limited, the high pH typical of sodic soils suggests that some salt-tolerant varieties may possess concomitant alkalinity resistance. A critical research gap remains the identification of hypogravity-adapted genotypes, for which publicly available data are presently absent. This represents a fundamental challenge requiring urgent investigation.

Key performance indicators for this stage include (1) identification of a minimum of three candidate varieties maintaining ≥80% viability under multifactorial stress simulations; (2) quantification of tolerance thresholds for critical parameters (e.g., radiation ≥0.67 mSv/day, pH 7.5–8.5, hypogravity 0.38 g); and (3) establishment of reproducible phenotypic markers for efficient high-throughput screening. These metrics will validate the progression of candidate genotypes to subsequent terrestrial simulation stages.

### 4.2. Stage 2 (Earth-Based Mars Simulation Training)

The work of Stage 1 lays the foundation for simulating Martian potato cultivation on Earth in this stage. The task in this stage is to grow potatoes under simulated Martian conditions. First, the varieties selected in Stage 1 are used to conduct separate planting experiments on Earth under simulated Martian environmental conditions, such as adjusting soil, water content, atmospheric composition, and temperature to be closer to natural Martian conditions while keeping other conditions the same as on Earth. Existing studies have shown that plants can maintain growth in Martian soil simulants, but current simulants may not fully replicate the physical and chemical properties of Martian soil (such as real mineral composition and toxic substance distribution), which requires further verification [1,23]. At the same time, it has been found that the gravity level on Mars can stimulate the gravitropic response of roots and maintain auxin polar transport [42].

Experimental progression is governed by quantifiable KPIs based on survival thresholds. If the survival rate is ≥60% in the middle of each simulated growth period, it is considered that the potato can adapt to this environmental condition, and the next stage can be considered. If the survival rate in a certain simulation experiment is ≤30%, the potato variety needs to be trained. The simulated Martian conditions in this experiment should be adjusted in several stages, from conditions close to Earth’s natural conditions to those close to Mars’ natural conditions. When the survival rate of potatoes in the experiment is ≥60%, the next stage can begin, gradually training the potato variety until it can adapt to the Martian natural conditions. It is expected that after 2–3 Martian seasons of experiments, relatively accurate simulation data can be obtained, and potato varieties that can adapt to various single natural conditions on Mars can be identified.

**Figure 4:**
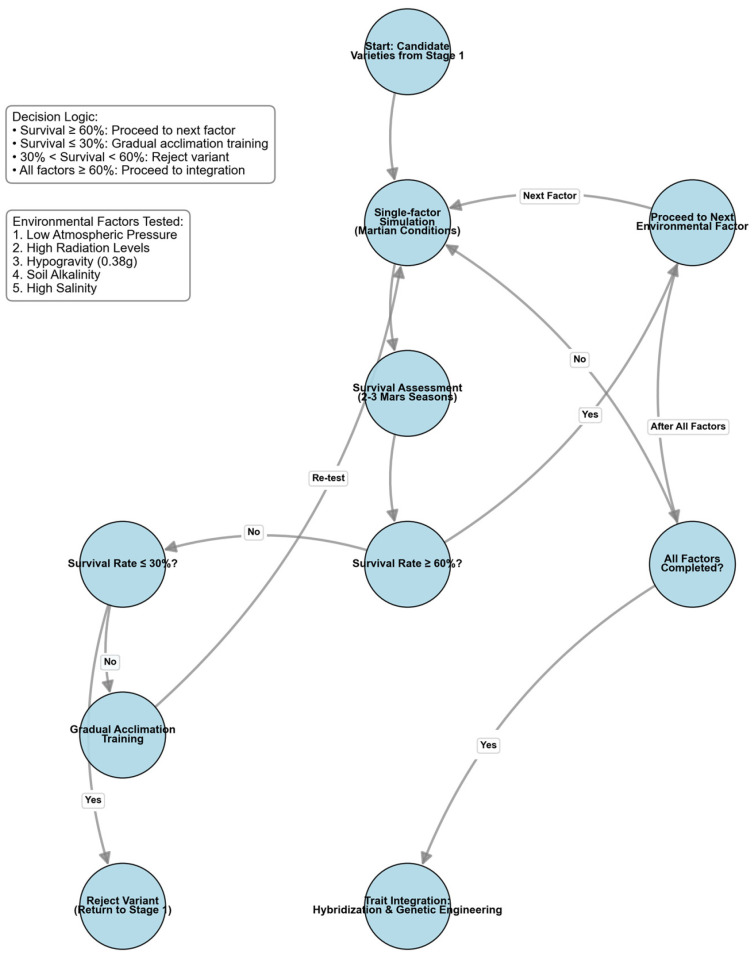
Flowchart of Potato Variety Screening and Domestication.

When the survival rate in all simulated Martian conditions is ≥60%, the excellent traits of the previously obtained potato varieties that are resistant to the harsh Martian environment should be integrated to the greatest extent through techniques such as hybridization, genetic engineering, and molecular engineering to ensure that the final potato variety can adapt to the Martian cultivation conditions after low-cost modification (Figure 4).

### 4.3. Stage 3 (Mars Base Planting)

The beginning of this stage marks the transformation of the Martian environment. The introduction of potatoes from Earth to Mars is similar to the introduction of crops from the south to the north on Earth and usually requires gradual regional adaptation adjustments. Therefore, the initial planting site of potatoes on Mars should be within the space base or the already established bioregenerative life support system. The potato varieties obtained in the second stage that can adapt to the modified Martian planting conditions should be introduced at this stage.

This stage focuses on deploying and operating a controlled cultivation module within a Martian habitat. This approach systematically addresses the seven fundamental challenges delineated in Section 3.1 (Biotic Challenges) and Section 3.2 (Abiotic Challenges), excluding the genetic adaptation limitations partially resolved in Stage 2. Our strategy employs targeted technological interventions to manage locally controllable parameters while establishing stable boundary conditions that compensate for globally immutable constraints. This framework ensures the maintenance of critical biological processes within a regulated environment, enabling sustainable potato cultivation. Key performance indicators for this stage include achieving ≥85% plant survival through complete growth cycles, maintaining ≥60% of Earth-based tuber yields, and establishing resource cycling systems with ≤15% dependency on external inputs per cultivation cycle.

#### 4.3.1. Nutrient Element Supply

Addressing the fundamental disruption to biogeochemical cycles, particularly the nitrogen and phosphorus limitations inherent in the sterile Martian regolith (as detailed in Section 3.1.1), constitutes the highest-priority biotic challenge. The proposed strategy moves beyond mere nutrient supplementation towards establishing a semi-closed, bioregenerative nutrient cycling system. This system is conceptualized to integrate in-situ resource utilization (ISRU) with the recycling of metabolic wastes from habitat operations, aiming to progressively reduce dependence on terrestrial imports.

The initial phase involves a rigorous characterization of the amended regolith’s geochemical properties. A comprehensive analysis would be essential to determine the baseline bioavailability of essential macro- and micronutrients (e.g., N, P, K, Ca, Mg, Fe, S) and to identify specific nutrient fixation or precipitation challenges, such as phosphorus insolubility at high pH. Following characterization, a dynamic nutrient management protocol is proposed. Instead of fixed application rates, this protocol would advocate for a feedback-controlled system based on real-time monitoring of soil nutrient status and plant tissue analysis. For instance, nutrient formulations could be adjusted phenologically, potentially shifting from a nitrogen-biased regimen (e.g., to support early vegetative growth) to a phosphorus- and potassium-enhanced formulation during tuber initiation and bulking. This approach acknowledges the need for adaptability in response to actual plant uptake and soil chemistry dynamics, which remain poorly constrained and subject to significant uncertainty.

A cornerstone of this conceptual system is the integration of organic waste streams. The processing of crew metabolic wastes (e.g., urine, feces) and food residues through controlled composting or anaerobic digestion is proposed as a means to close the nutrient loop. The resulting organic amendments could serve dual purposes: partially replenishing essential nutrients like nitrogen and phosphorus and contributing to the development of a soil organic matrix capable of improving cation exchange capacity and nutrient retention. However, the mass, energy, and sanitation requirements for establishing and maintaining such bioreactor facilities present significant logistical challenges that must be quantified in future mission architectures.

Ultimately, the envisioned nutrient cycling system aims for a synergistic integration of slow-release organic nutrients from in-situ processing with precision-delivered, quick-release inorganic supplements. This hybrid strategy is postulated to more closely mimic terrestrial nutrient dynamics, potentially enhancing nutrient use efficiency and soil health over the long term. The success of this conceptual framework hinges on future research to model nutrient fluxes, quantify the resource penalties of waste processing, and validate the efficacy and stability of these cycles under simulated Martian conditions.

#### 4.3.2. Microbial Ecosystem Synthesis

Addressing the profound challenge of a sterile regolith and the absence of a functional soil microbiome (Section 3.1.2) is critical for achieving sustainable nutrient cycling and plant health. The proposed conceptual framework moves beyond simple inoculation towards the deliberate synthesis of a minimal, resilient microbial ecosystem. This ecosystem is designed to perform core functions: nutrient transformation (e.g., nitrogen fixation, phosphorus solubilization), pathogen suppression, and contribution to soil aggregate formation. The strategy involves a phased introduction of microbial consortia and, potentially, soil fauna, contingent upon rigorous assessment of planetary protection protocols and system stability.

The initial inoculum would be composed of microbial agents selected for multi-stress tolerance, particularly to salinity and alkalinity. This could include specific strains of nitrogen-fixing bacteria (e.g., Azotobacter), phosphate-solubilizing bacteria (e.g., Pseudomonas), and symbiotic fungi like Trichoderma, which can also exhibit antagonistic activity against pathogens. The inoculation ratios and densities (e.g., a suggested initial ratio of 1:50 for carrier to soil) are presented as starting parameters for experimental validation, as their optimal values are expected to shift significantly under Martian conditions. A critical dependency for microbial establishment is the provision of a labile carbon source. The use of processed organic wastes (e.g., 5% bio-organic fertilizer from composted inedible biomass) is conceptualized as a primary strategy, though its production scale and consistency represent a significant logistical challenge.

To enhance the adaptation potential of the soil community, a systematic screening of extremotolerant microorganisms from Earth’s analog environments—such as polar regions, hyper-arid deserts, and high-altitude soils—is proposed [43]. These environments subject microbes to polyextremes, including desiccation, hypersalinity, oligotrophy, and high UV radiation, which collectively serve as a proxy for pre-adaptation to Martian stresses. Halophilic archaea from saline lakes maintain functionality under high ionic strength, a relevant trait for managing the saline Martian regolith. These candidate strains would be subjected to incremental exposure to simulated Martian conditions (e.g., low pressure, high CO_2_, radiation) to identify the most robust genotypes. Subsequent co-culture experiments would aim to develop synergistic consortia that mimic the functional redundancy of terrestrial ecosystems. The cultivation and expansion of these beneficial microorganisms into applicable inoculants is a critical step. A proposed application rate of 100–200 g per cubic meter of regolith, amended with organic matter, should be viewed as a baseline for testing colonization efficiency, recognizing that the ultimate survival and functionality of these communities remain a major uncertainty and a key focus for future research.

The introduction of soil fauna, such as the earthworm Eisenia fetida, is contemplated for its potential role in organic matter decomposition, soil aeration, and even perchlorate degradation via its gut enzyme systems. However, the mass, life support, and ecological control requirements for maintaining macrofauna, along with their potential to disrupt carefully managed microbial niches, present substantial hurdles that must be thoroughly evaluated.

Long-term system viability would depend on continuous monitoring and adaptive management. A soil microbial monitoring system, leveraging molecular techniques like quantitative PCR and high-throughput sequencing, is proposed to track community succession and functional gene abundance [44,45]. This data would inform a feedback control system designed to maintain ecological balance. For example, if monitoring indicates a rise in potential phytopathogens, the system could trigger the introduction of specific biocontrol agents or adjust environmental parameters to disfavor the pathogen. Conversely, a decline in key functional groups, such as nitrifiers, would prompt the supplemental inoculation of targeted microbial agents. Agronomic practices such as crop rotation or fallow periods are also considered as potential tools to manage soil health and prevent microbial community degeneration. The success of this entire conceptual framework hinges on future empirical research to determine the stability, resilience, and functional output of synthesized microbial ecosystems under simulated Martian conditions, while strictly adhering to planetary protection principles.

#### 4.3.3. Hydro-Ecological System Integration

Addressing the hydro-ecological integration challenges outlined in Section 3.1.3—specifically, the extreme aridity, limited water retention capacity of the regolith, and associated physiological stresses—requires a closed-loop water management strategy. This approach aims to precisely match water availability with plant demand while minimizing losses, acknowledging the significant energy and mass constraints of water extraction and recycling on Mars.

The proposed concept centers on an intelligent irrigation system that synergistically combines low-volume delivery methods. A subsurface drip irrigation component is envisioned to deliver water directly to the root zone, maximizing uptake efficiency and minimizing evaporative losses in the dry atmospheric conditions. This could be supplemented by an ultrasonic fogging system, activated during specific phenological phases to elevate ambient humidity transiently, thereby potentially reducing plant transpirational demand and mitigating vapor pressure deficit stress. The integration of a sensor network for real-time monitoring of soil water potential, substrate moisture content, and leaf turgor pressure is considered fundamental for enabling feedback control. Such a system would aim to schedule irrigation based on actual plant physiological needs rather than fixed timetables, with the goal of achieving high water-use efficiency. While some terrestrial studies indicate that such optimized systems can potentially increase water use efficiency by 60–70% [46], the actual performance under Martian conditions remains unvalidated and subject to significant uncertainty, requiring rigorous modeling and prototyping.

The ultimate sustainability of the water cycle is conceptually tied to in-situ resource utilization (ISRU). The existence of subsurface water ice, particularly at high latitudes [47,48,49], is noted as a potential source. However, the feasibility of this source is contingent upon the development of extraction and purification technologies whose energy demands and implementation logistics are substantial and not yet quantified for a Martian agricultural context. The proposed water management system is therefore framed as a critical, yet highly challenging, component whose design must be co-optimized with the overall energy and mass budget of a future habitat.

#### 4.3.4. Atmospheric Environment Control

The profound disparities in atmospheric pressure and composition between Mars and terrestrial conditions (Section 3.2.1, Table 1) represent absolute boundary constraints, necessitating a fully enclosed and pressurized cultivation module. The conceptual design for this module prioritizes the maintenance of a physiologically compatible atmosphere through integrated physicochemical systems.

The foundational element is a pressurized growth habitat. A potential design involves a modular structure employing materials such as ethylene-tetrafluoroethylene (ETFE) copolymer, which is considered for its high light transmittance and durability [50,51]. The internal pressure would be maintained above the critical threshold for potato physiology (≥50 kPa) to prevent stomatal malfunction and support normal gas exchange processes.

Atmospheric composition within the module is actively managed. The high ambient CO_2_ concentration (95.3%) is viewed as a resource that can be scrubbed to optimal photosynthetic levels (approximately 0.03–0.05%) using adsorption technologies, such as zeolite molecular sieves [52,53,54]. Concurrently, molecular oxygen, critical for root respiration and metabolic functions, must be replenished. A proposed method involves the electrolysis of water, potentially sourced from recycled crew wastewater [55], to generate oxygen and contribute to gas balance. Mechanical ventilation, achieved with low-power circulation systems, is considered essential to ensure homogeneous gas distribution and minimize stagnant boundary layers around plant leaves, thereby optimizing CO_2_ diffusion for photosynthesis [56]. The integration and energy demands of these life support subsystems present a significant engineering challenge that must be co-optimized with the habitat’s overall power budget.

#### 4.3.5. Thermodynamic Regulation

Addressing the extreme thermal environment of Mars, characterized by an average temperature of −63 °C and diurnal fluctuations exceeding 120 °C (Section 3.2.2), requires a robust thermal regulation strategy for the cultivation module. The proposed approach focuses on minimizing heat loss and stabilizing root-zone temperatures through passive and active insulation concepts.

A key concept involves the use of composite insulating layers to decouple the internal soil temperature from the external radiative environment. This could incorporate a base layer of highly porous material like expanded perlite, noted for its low thermal conductivity (~0.04 W/m·K) [57,58,59], to reduce conductive heat loss. Supplementing this with a surface layer of biochar particles is theorized to provide additional insulation and potentially improve soil properties over time [56]. The combination of such materials aims to create a thermal buffer, reducing the energy penalty associated with maintaining a suitable root-zone temperature. While such composite structures have been studied in terrestrial contexts [60], their performance under Martian conditions—particularly in mitigating radiative heat loss—is unproven. The claim of a 60% reduction in heat loss and the stabilization of soil temperature at 8–10 °C is presented as a potential performance target based on terrestrial analogs; its actual achievement on Mars is subject to validation and depends heavily on the specific engineering implementation and the severe external thermal gradient.

The primary challenge lies in the significant energy required to maintain thermal equilibrium against the extreme cold. The feasibility of this conceptual design is therefore contingent upon the development of highly efficient insulation materials and a reliable energy source, the mass and power requirements of which remain major constraints for mission architecture planning.

#### 4.3.6. Pedological Amendment and Geochemical Management

This section addresses the pedological constraints and geochemical limitations critical for plant growth, as detailed in Section 3.2.3. The strategy focuses on transforming the inert, alkaline Martian regolith into a functional growth medium through a multi-pronged conceptual approach that prioritizes in-situ resource utilization (ISRU) and long-term sustainability, while acknowledging the significant mass and energy constraints of large-scale soil construction.

The primary objective is to initiate a pedogenetic process. A proposed method involves the strategic layering of mechanically crushed and graded local regolith to create a more structured soil profile, mimicking terrestrial soil horizons. The incorporation of locally sourced clay minerals (e.g., montmorillonite, kaolinite), if available, is considered for their potential to improve cation exchange capacity and water-holding characteristics. The development of a soil organic matrix is seen as a long-term goal, theoretically achievable through the continuous addition and decomposition of organic wastes, including processed crew wastes and inedible biomass. However, the timescales, rates of humification, and the vast quantities of organic matter required present a formidable logistical challenge that must be quantified in future mission architectures.

A major focus is managing the inherent alkalinity (pH ~7.5–8.5), which induces phosphorus fixation and nutrient imbalances. The proposal explores the use of acidic amendments, such as elemental sulfur, which could theoretically acidify the regolith through microbial oxidation. The concept of creating an integrated pH-buffering system is also introduced. This could involve amending the regolith with minerals possessing intrinsic buffering capacity or by embedding slow-release amendments to stabilize the rhizosphere pH. The addition of organic substances with buffering capacity, such as humic acids, is also contemplated for their dual role in pH modulation and microbial support. These approaches present significant challenges in terms of the mass, sourcing, and delivery of amendments, underscoring the need for ISRU-focused mineral processing technologies. Quantitative claims regarding improvements in water retention or porosity are presented as potential outcomes based on terrestrial analogies, the realization of which under Martian conditions is highly uncertain and subject to experimental validation.

The overall strategy is framed as a phased bio-geoengineering effort. The initial phase would rely more heavily on direct amendment and conditioning. The long-term vision is to foster a progressively self-sustaining system where biological activity (Section 4.3.2) and nutrient cycling (Section 4.3.1) contribute significantly to maintaining soil health. The viability of this entire framework is contingent upon future research to quantify the mass and energy budgets of regolith processing and to validate the proposed amendment strategies in relevant analog environments.

#### 4.3.7. Managing Radiation and Hypogravity Constraints

This section addresses the globally immutable parameters of radiation and gravity (Section 3.2.4), which constitute fixed boundary conditions that cannot be engineered at a planetary scale. The proposed strategies are therefore confined to protective mitigation within the cultivated volume.

For ionizing radiation, a primary challenge is shielding against galactic cosmic rays and solar particle events. Conceptual shielding approaches could involve a combination of mass, such as water or other hydrogen-rich polymers [61,62], and specialized materials [63,64]. However, the practicality, mass penalty, and potential secondary radiation effects of any shielding material require rigorous engineering analysis and trade-off studies against the alternative of locating cultivation modules underground or behind substantial regolith barriers.

Regarding hypogravity (0.38 g), the physiological implications for potato growth remain a fundamental research question. The strategy emphasizes selecting and engineering genotypes (as initiated in Stage 1) for adaptability to altered gravitropism and hydrostatic pressures. Beyond genetic adaptation, environmental parameters such as light spectra, photoperiod, and temperature regimes can be carefully controlled to optimize growth and tuberization, potentially compensating for some gravitational effects. The focus is on managing the growth environment to support plant development within this immutable physical constraint.

The sequential addressing of the outlined challenges, from nutrient cycling and microbial synthesis to atmospheric control and pedological management, conceptualizes a pathway toward achieving stable potato cultivation under Martian conditions. This phased approach envisions that through iterative cycles of cultivation within a controlled base habitat, the reliance on external inputs could be progressively reduced, enhancing system closure over time. The integration of targeted technological interventions with biological adaptation—particularly for immutable constraints like radiation and hypogravity—constitutes a foundational challenge for future habitat design. The development and validation of this integrated system present a complex, multi-decade research endeavor critical to advancing toward sustainable agricultural systems on Mars.

### 4.4. Stage 4 (Mars Environment Planting)

The successful establishment of controlled cultivation within Martian habitats (Stage 3) enables consideration of transition pathways toward extra-habitat agricultural systems. This stage represents a conceptual exploration of potential expansion scenarios, acknowledging the profound technical and biological challenges inherent in open-environment cultivation on Mars. Key performance indicators for this stage include (1) achieving ≥70% plant survival rate during initial acclimatization cycles; (2) maintaining tuber yields at ≥40% of Earth-based controls under partial environmental regulation; and (3) demonstrating progressive reduction in habitat energy inputs by ≥25% per growth cycle.

Implementation requires systematic environmental modification beyond habitat confines, referencing soil remediation protocols established in Stage 3. The transition strategy employs gradual acclimatization through partially shielded enclosures, with flexibility to adjust protocols based on real-time performance data. While Stage 3 developed varieties tolerant to hypogravity and low-pressure environments, the extreme conditions of open Martian exposure necessitate careful risk assessment and adaptive management approaches to address unforeseen physiological challenges.

## 5. Conclusions

This study has formulated a structured, hierarchical framework to assess the feasibility of potato cultivation on Mars, advancing the discourse beyond qualitative challenges toward a quantitative, hypothesis-driven research agenda. The primary contribution is a four-stage conceptual roadmap, defined not by prescriptive engineering solutions but by testable hypotheses and stage-gated KPIs. This represents a novel synthesis, integrating discrete challenges in plant physiology, soil science, and systems engineering into a coherent, phased development logic that prioritizes interventions according to parameter modifiability and physiological criticality.

Compared to existing literature focusing on isolated stressors or generic life support concepts, this framework introduces a systematic methodology for stratifying and prioritizing challenges (biotic/abiotic, high/medium/low criticality) and linking them to specific validation milestones. However, the analysis explicitly delineates the boundaries of current feasibility. Several components of the roadmap, particularly in later stages, remain conceptual propositions rather than foreseeable implementations. The globally immutable constraints of Martian radiation, gravity, and atmospheric pressure necessitate fully engineered habitats, making any form of unshielded, open-field agriculture infeasible with foreseeable technology. Furthermore, the mass, energy, and logistical burdens of large-scale soil amendment, water provisioning, and infrastructure deployment present prohibitive challenges that are acknowledged but not resolved within this conceptual study.

The proposed framework intentionally generates actionable research questions. Future work must transition from concept to quantitative validation, focusing on: (1) Establishing terrestrial analog experiments to test the defined KPIs for Stage 1 (e.g., genotype survival under multifactorial stress) and Stage 2 (e.g., yield thresholds in simulated integrated environments); (2) Systems Modeling and Trade-off Analyses: Developing integrated mass, energy, and cost models to quantify the resources required for regolith processing, closed-loop system operation, and scaling, moving beyond qualitative design; (3) Biosafety and Adaptation Research: Investigating confined ecosystem dynamics and plant–microbe interactions under Mars-like conditions, explicitly within planetary protection guidelines, to address the viability of introducing terrestrial biology.

In summary, this work provides a necessary foundational framework and a prioritized research agenda for Martian agriculture. It clarifies that the path forward lies not in seeking a single breakthrough but in executing a coordinated sequence of targeted experiments and analyses, each designed to rigorously test a defined hypothesis within this structured roadmap.

## Figures and Tables

**Table 1:** Synthesis of Parametric Disparities Between Martian Conditions and Potato Cultivation Prerequisites.

Parameter	Martian Conditions	Potato Growth Requirements & Limits
**Atmospheric Pressure**	~630 Pa [10]	Requires 18–21% O_2_ at ~101.325 kPa; stomatal malfunction < 50 kPa; tuber inhibition at soil O_2_ < 18 kPa.
**Atmospheric Composition**	95.3% CO_2_, 2.7% N_2_, 1.6% Ar [11]	Optimal CO_2_: 0.03–0.05%; requires adequate O_2_ (18–21%).
**Surface Temperature**	Average: −63 °C; Diurnal Δ > 120 °C; poles down to −153 °C [12]	Optimal: 25.6–29.8 °C [13]; activity ceases < 5 °C; hindered > 35 °C; optimal diurnal Δ 8–12 °C [14,15]. Root development requires ≥ 7 °C; frost damage at −2 °C.
**Water Availability**	Precipitable H_2_O: max ~0.1 mm (N. summer), ~0.05 mm (S. hemisphere), <0.005 mm (low latitudes) [16]; exists as polar ice caps [17,18]; potential brine seepage [16,17,18,19,20].	Requirement: 500–700 mm/cycle [19]; root zone at 24–38 cm depth, field capacity 60–80% [20]; moisture < 40% reduces starch synthesis enzymes > 50%.
**Soil pH**	Alkaline, pH 7.5–8.5 [21,22,23,24]	Optimal range: 5.0–6.8 [25,26,27,28].
**Soil Structure/Porosity**	Fine particles, low porosity, poor aeration, low water-stable aggregates (<15%), bulk density 1.6–1.8 g/cm^3^ [1,28,29]	Requires porosity > 50%, bulk density 1.0–1.3 g/cm^3^, water-stable aggregates (>40%, diameter 0.25–2 mm) for O_2_ diffusion rate (ODR) ≥ 30 μg/cm^2^/min.
**Nutrient Elements**	Contains essential (C, H, O, N, P, S, K, Mg, Na, Ca) and trace elements (Mn, Ni, Mo, Cu, Fe, Zn) but with low bioavailability [25,26,27]; Total N < 0.1%; high Cl content (0.5–1.0 wt%) [22].	Requires 4–6 kg N, 0.8–1.5 kg P, 6–8 kg K per ton of tubers [29]; soil NO_3_^−^-N/NH_4_^+^-N ratio 3:1 [30]; available K > 120 mg/kg [31]; B: 0.5–1.0 mg/kg, Zn: 1.5–2.0 mg/kg [32].
**Soil Microbiology**	Lacks organic carbon (TOC < 0.1%) [33], leading to negligible microbial activity.	Requires active microbiome and organic matter (≥2%) for nutrient cycling.
**Radiation**	~0.67 mSv/day (annual 244.55 mSv) [34].	Earth’s annual dose ~0.3 mSv.
**Gravity**	0.38 g [35,36].	Adapted to 1.0 g.

## Data Availability

No new data were created or analyzed in this study.

## Abbreviations

The following abbreviations are used in this manuscript:

BLSS	Bioregenerative Life Support Systems
KPIs	Key Performance Indicators

## References

[1] Wamelink G.W.W., Frissel J.Y., Krijnen W.H.J., Verwoert M.R., Goedhart P.W. (2014). Can plants grow on mars and the moon: A growth experiment on mars and moon soil simulants. PLoS ONE.

[2] Ramírez D.A., Kreuze J., Amoros W., Valdivia-Silva J.E., Ranck J., Garcia S., Salas E., Yactayo W. (2017). Extreme salinity as a challenge to grow potatoes under Mars-like soil conditions: Targeting promising genotypes. Int. J. Astrobiol..

[3] Perchonok M.H., Cooper M.R., Catauro P.M., Doyle M.P., Klaenhammer T.R. (2012). Mission to Mars: Food Production and Processing for the Final Frontier. Annual Review of Food Science and Technology.

[4] Wheeler R.M. (2017). Agriculture for Space: People and Places Paving the Way. Open Agric..

[5] Birch P.R.J., Bryan G., Fenton B., Gilroy E.M., Hein I., Jones J.T., Prashar A., Taylor M.A., Torrance L., Toth I.K. (2012). Crops that feed the world 8: Potato: Are the trends of increased global production sustainable?. Food Secur..

[6] Zimmerer K.S. (1998). The Ecogeography of Andean Potatoes. BioScience.

[7] Wheeler R.M. (2006). Potato and Human Exploration of Space: Some Observations from NASA-Sponsored Controlled Environment Studies. Potato Res..

[8] Woolfe J.A. (1986). Potato—A gift from the Andes. Nutr. Bull..

[9] Renault D., Wallender W.W. (2000). Nutritional water productivity and diets. Agric. Water Manag..

[10] de Pater I., Lissauer J.J. (2015). Planetary Sciences.

[11] Mahaffy P.R., Webster C.R., Atreya S.K., Franz H., Wong M., Conrad P.G., Harpold D., Jones J.J., Leshin L.A., Manning H. (2013). Abundance and Isotopic Composition of Gases in the Martian Atmosphere from the Curiosity Rover. Science.

[12] Apéstigue Palacio V. (2019). Diseño de Un Radiómetro Miniaturizado Para La Exploración de Marte. Ph.D. Thesis.

[13] Matusiak M. (2020). Features of Growth and Development of the Genus Forsythia Vahl. In Conditions of the Vinnytsia National Agrarian University Biostationary. Agric. For..

[14] Aber J.D., Reich P.B., Goulden M.L. (1996). Extrapolating leaf CO_2_ exchange to the canopy: A generalized model of forest photosynthesis compared with measurements by eddy correlation. Oecologia.

[15] Khan A., Khan V., Pandey K., Sopory S.K., Sanan-Mishra N. (2022). Thermo-Priming Mediated Cellular Networks for Abiotic Stress Management in Plants. Front. Plant Sci..

[16] Smith M.D. (2002). The annual cycle of water vapor on Mars as observed by the Thermal Emission Spectrometer. J. Geophys. Res. Planets.

[17] Carr M.H., Head J.W. (2015). Martian surface/near-surface water inventory: Sources, sinks, and changes with time. Geophys. Res. Lett..

[18] Plaut J.J., Picardi G., Safaeinili A., Ivanov A.B., Milkovich S.M., Cicchetti A., Kofman W., Mouginot J., Farrell W.M., Phillips R.J. (2007). Subsurface radar sounding of the south polar layered deposits of Mars. Science.

[19] Allen R., Pereira L., Raes D., Smith M., Allen R.G., Pereira L.S., Martin S. (1998). Crop Evapotranspiration: Guidelines for Computing Crop Water Requirements.

[20] Zhang Y., Han K., Jung K., Cho H., Seo M., Sonn Y. (2017). Study on the Standards of Proper Effective Rooting Depth for Upland Crops. Korean J. Soil Sci. Fertil..

[21] Day J.M.D. (2009). Planetary crusts: Their composition, origin and evolution, by Stuart Ross Taylor and Scott M. McLennan. Meteorit. Planet. Sci..

[22] Hecht M.H., Kounaves S.P., Quinn R.C., West S.J., Young S.M.M., Ming D.W., Catling D.C., Clark B.C., Boynton W.V., Hoffman J. (2009). Detection of perchlorate and the soluble chemistry of martian soil at the Phoenix lander site. Science.

[23] Kasiviswanathan P., Swanner E.D., Halverson L.J., Vijayapalani P. (2022). Farming on Mars: Treatment of basaltic regolith soil and briny water simulants sustains plant growth. PLoS ONE.

[24] McSween H.Y., Taylor G.J., Wyatt M.B. (2009). Elemental Composition of the Martian Crust. Science.

[25] Jasim A., Sharma L.K., Zaeen A., Bali S.K., Buzza A., Alyokhin A. (2020). Potato Phosphorus Response in Soils with High Value of Phosphorus. Agriculture.

[26] Mandisi M.M., Ngaka S.G., Oghenetsavbuko E.T. (2016). Effects of Different Soil Amendments on Soil pH and Heavy Metals Content in Maize (*Zea mays* [L.]). Agric. For. Fish..

[27] Nurza I.S.A. (2020). Uji Kelayakan Tanah terhadap Penanaman Tanaman Pisang, Singkong, dan Ubi Jalar di Daerah Sekitar Vila Silma Kecamatan Cilember Kabupaten Bogor. Risenologi.

[28] Xing Y., Niu X., Wang N., Jiang W., Gao Y., Wang X. (2020). The Correlation between Soil Nutrient and Potato Quality in Loess Plateau of China Based on PLSR. Sustainability.

[29] Koch M., Naumann M., Pawelzik E., Gransee A., Thiel H. (2019). The Importance of Nutrient Management for Potato Production Part I: Plant Nutrition and Yield. Potato Res..

[30] Meng F., Zhang R., Zhang Y., Li W., Zhang Y., Zhang M., Yang X., Yang H. (2024). Improving maize carbon and nitrogen metabolic pathways and yield with nitrogen application rate and nitrogen forms. PeerJ.

[31] Yuningsih L., Bastoni B., Yulianty T., Harbi J. (2019). Sifat Fisika Dan Kimia Tanah Pada Lahan Hutan Gambut Bekas Terbakar: Studi Kasus Kabupaten Ogan Komering Ilirsumatera Selatan, Indonesia Physical and Chemical Properties of Burnt Peat Land Forest: Case Study in Ogan Komering Ilir Regency, South Sumatra, Indonesia. Sylva J. Ilmu-Ilmu Kehutan..

[32] EL-Anany A.M.A., Anany T.G. (2020). Effect of some mineral nutrients on productivity, tuber seed quality and storability of Jerusalem artichoke. Middle East J. Agric. Res..

[33] Plane J.M.C., Flynn G.J., Määttänen A., Moores J.E., Poppe A.R., Carrillo-Sanchez J.D., Listowski C. (2017). Impacts of Cosmic Dust on Planetary Atmospheres and Surfaces. Space Sci. Rev..

[34] Jungmann F., Bila T., Kleinert L., Mölleken A., Möller R., Schmidt L., Schneider N., Teiser J., Utzat D., Volkenborn V. (2021). Cosmic radiation does not prevent collisional charging in (pre)-planetary atmospheres. Icarus.

[35] Dudley-Flores M., Gangale T. (2007). Globalization of Space—The Astrosociological Approach. Proceedings of the AIAA SPACE 2007 Conference & Exposition.

[36] Leconte J. (2021). Spectral binning of precomputed correlated-k coefficients. Astron. Astrophys..

[37] Tokuda A., Kitaya Y. (2019). Effects of gravity direction on water transport in sweetpotato plants. Biol. Sci. Space.

[38] Clark B.C., Van Hart D.C. (1981). The salts of Mars. Icarus.

[39] Li Q., Qin Y., Hu X., Jin L., Li G., Gong Z., Xiong X., Wang W. (2022). Physiology and Gene Expression Analysis of Potato (*Solanum tuberosum* L.) in Salt Stress. Plants.

[40] Rahman M.H., Deepo D.M., Islam M.M., Bashar M.A., Sheuly K.N., Syfullah K., Hoque M.E., Molla M.M.H. (2023). In Vitro Shoot Bioassay of Salt Tolerant International Potato Center Bred Potato Genotypes for Assessing Their Salinity Tolerance. Turk. J. Agric. Food Sci. Technol..

[41] Tufan Ü., Öztürk E. (2024). Growth, Yield Components and Tuber Yield Responses of Potato (*Solanum tuberosum* L.) Varieties in High Altitude Regions of Türkiye. J. Agric. Prod..

[42] Medina F.J., Manzano A., Villacampa A., Ciska M., Herranz R. (2021). Understanding Reduced Gravity Effects on Early Plant Development Before Attempting Life-Support Farming in the Moon and Mars. Front. Astron. Space Sci..

[43] Zhang Q., White J.F. (2021). Bioprospecting Desert Plants for Endophytic and Biostimulant Microbes: A Strategy for Enhancing Agricultural Production in a Hotter, Drier Future. Biology.

[44] Barea J.M. (2015). Future challenges and perspectives for applying microbial biotechnology in sustainable agriculture based on a better understanding of plant-microbiome interactions. J. Soil Sci. Plant Nutr..

[45] Evdokimova E., Ivanova E., Gladkov G., Zverev A., Kimeklis A., Serikova E., Pinaev A., Kichko A., Aksenova T., Andronov E. (2024). Structural Shifts in the Soil Prokaryotic Communities Marking the Podzol-Forming Process on Sand Dumps. Soil Syst..

[46] Sahin H. (2024). A Cheap and Basic Solar-Powered Smart Irrigation System Proposal for Medium and Small-Scale Farming. Eur. J. Eng. Technol. Res..

[47] Read P.L., Lewis S.R., Mulholland D.P. (2015). The physics of Martian weather and climate: A review. Rep. Prog. Phys..

[48] Zhao Y.-Y.S., Zhou D., Li X., Liu J., Wang S., Ouyang Z. (2020). The evolution of scientific goals for Mars exploration and future prospects. Chin. Sci. Bull. Chin..

[49] Zheng N., Ding C., Su Y., Orosei R. (2024). Water Ice Resources on the Shallow Subsurface of Mars: Indications to Rover-Mounted Radar Observation. Remote Sens..

[50] Park D.Y., Lee H.J., Yun S.I., Choi S.M. (2021). Simulation Analysis of Daylight Characteristics and Cooling Load Based on Performance Test of Covering Materials Used in Smart Farms. Energies.

[51] Surholt F., Uhlemann J., Stranghöner N. (2022). Temperature and Strain Rate Effects on the Uniaxial Tensile Behaviour of ETFE Foils. Polymers.

[52] Mesfer M.K.A., Danish M., Khan M.I., Ali I.H., Hasan M., Jery A.E. (2020). Continuous Fixed Bed CO_2_ Adsorption: Breakthrough, Column Efficiency, Mass Transfer Zone. Processes.

[53] Parinyakit S., Worathanakul P. (2021). Static and Dynamic Simulation of Single and Binary Component Adsorption of CO_2_ and CH_4_ on Fixed Bed Using Molecular Sieve of Zeolite 4A. Processes.

[54] Salmasi M., Fatemi S., Doroudian Rad M., Jadidi F. (2013). Study of carbon dioxide and methane equilibrium adsorption on silicoaluminophosphate-34 zeotype and T-type zeolite as adsorbent. Int. J. Environ. Sci. Technol..

[55] Wang L., Zhang K., Yuan L., Guo J., Tian Y. (2024). Design of a novel wastewater reuse system for space station based on urea electrooxidation technology. J. Northwestern Polytech. Univ..

[56] Osman A.I., Farghali M., Dong Y., Kong J., Yousry M., Rashwan A.K., Chen Z., Al-Fatesh A., Rooney D.W., Yap P.-S. (2024). Reducing the carbon footprint of buildings using biochar-based bricks and insulating materials: A review. Environ. Chem. Lett..

[57] Kumar R., Srivastava A., Lakhani R. (2022). Industrial Wastes-Cum-Strength Enhancing Additives Incorporated Lightweight Aggregate Concrete (LWAC) for Energy Efficient Building: A Comprehensive Review. Sustainability.

[58] Maaloufa Y., Mounir S., Khabbazi A., Kettar J. (2016). Effect of Calcination on the Thermal Properties of Bricks Done From Clay-Expanded Perlite on Insulating Walls. Res. J. Appl. Sci. Eng. Technol..

[59] Politi C., Peppas A., Taxiarchou M. (2023). Data-Driven Integrated Decision Model for Analysing Energetic Behaviour of Innovative Construction Materials Capable of Hybrid Energy Storage. Sustainability.

[60] Faleh S.T., Abood M.A., Fahmi A.H. (2023). The Role of Biochar and Perlite in Improving some Physical Properties of Clay Loam and Sandy Loam Soil. IOP Conf. Ser. Earth Environ. Sci..

[61] Tsyats’ko V.V., Gokov S.P., Kazarinov Y.G. (2023). Interaction of Fluxes of Fast and Thermal Neutrons with an Aqueous Solution of Organic Dye Methylene Blue Containing and not Containing Boric Acid.

[62] Uğur F.A. (2017). New applications and developments in the neutron shielding. EPJ Web Conf..

[63] Mutlak D.A., Ali A.H. (2013). Study the enhancement of the radiation shielding afforded to epoxy/lead composites. J. Univ. Anbar Pure Sci..

[64] Li Z., Zhou W., Zhang X., Gao Y., Guo S. (2021). High-efficiency, flexibility and lead-free X-ray shielding multilayered polymer composites: Layered structure design and shielding mechanism. Sci. Rep..

